# Reconstruction of White Matter Tracts via Repeated Deterministic Streamline Tracking – Initial Experience

**DOI:** 10.1371/journal.pone.0063082

**Published:** 2013-05-06

**Authors:** Miriam H. A. Bauer, Daniela Kuhnt, Sebastiano Barbieri, Jan Klein, Andreas Becker, Bernd Freisleben, Horst K. Hahn, Christopher Nimsky

**Affiliations:** 1 Department of Neurosurgery, University of Marburg, Marburg, Germany; 2 Department of Mathematics and Computer Science, University of Marburg, Marburg, Germany; 3 International Clinical Research Center, St. Anne’s University Hospital Brno, Brno, Czech Republic; 4 Fraunhofer MEVIS - Institute for Medical Image Computing, Bremen, Germany; University of Cambridge, United Kingdom

## Abstract

Diffusion Tensor Imaging (DTI) and fiber tractography are established methods to reconstruct major white matter tracts in the human brain in-vivo. Particularly in the context of neurosurgical procedures, reliable information about the course of fiber bundles is important to minimize postoperative deficits while maximizing the tumor resection volume. Since routinely used deterministic streamline tractography approaches often underestimate the spatial extent of white matter tracts, a novel approach to improve fiber segmentation is presented here, considering clinical time constraints. Therefore, fiber tracking visualization is enhanced with statistical information from multiple tracking applications to determine uncertainty in reconstruction based on clinical DTI data. After initial deterministic fiber tracking and centerline calculation, new seed regions are generated along the result’s midline. Tracking is applied to all new seed regions afterwards, varying in number and applied offset. The number of fibers passing each voxel is computed to model different levels of fiber bundle membership. Experimental results using an artificial data set of an anatomical software phantom are presented, using the Dice Similarity Coefficient (DSC) as a measure of segmentation quality. Different parameter combinations were classified to be superior to others providing significantly improved results with DSCs of 81.02%±4.12%, 81.32%±4.22% and 80.99%±3.81% for different levels of added noise in comparison to the deterministic fiber tracking procedure using the two-ROI approach with average DSCs of 65.08%±5.31%, 64.73%±6.02% and 65.91%±6.42%. Whole brain tractography based on the seed volume generated by the calculated seeds delivers average DSCs of 67.12%±0.86%, 75.10%±0.28% and 72.91%±0.15%, original whole brain tractography delivers DSCs of 67.16%, 75.03% and 75.54%, using initial ROIs as combined include regions, which is clearly improved by the repeated fiber tractography method.

## Introduction

Multimodal navigation guidance is a routine tool in neurosurgical operating theaters to achieve best possible resection of the lesion with minimum postoperative morbidity, displaying outlines of the segmented objects in the microscope heads-up display. In addition to mere anatomical magnetic resonance imaging (MRI) data, the multimodality concept includes the visualization of functional brain areas with cortical sites (functional MRI, magneto-encephalography), fiber bundles (diffusion tensor imaging (DTI) based fiber tractography) or metabolic data (single photon emission computed tomography, positron emission tomography, magnetic resonance spectroscopy imaging). To date, the concept of maximum resection whilst preserving neurological functions is not only self-evident for benign lesions, but also for malignancies such as gliomas, as the most common primary brain tumors [1]. Whereas the correlation of their extent of resection (EOR) and patient outcome has been a long-term point of discussion, recent literature favors also radical EOR in surgery of low-grade and high-grade gliomas [1]–[5]. Another major addition to multimodal navigation is intraoperative MRI (iMRI) followed by an update of the navigation to compensate for the effects of brain shift (brain deformations due to e.g. loss of cerebrospinal fluid, tumor resection, edema) [6]–[9]. It was demonstrated that iMRI combined with navigation guidance and an intraoperative update of image data leads to higher rates of EOR and gross total resection rates in glioma surgery with low postoperative morbidity [10]–[13].

In order to maximize the extent of tumor volume resection whilst preserving neurological functions, additional image data sets can be used to display more information on the tumor or risk structures such as vessels, subcortical neuronal pathways or eloquent cortical areas. In this paper, we focus on the reconstruction and visualization of subcortical fiber bundles, delivered by DTI and fiber tractography.

DTI has first been described by Basser et al. [14] in 1994. Upon this finding, DTI based fiber tractography became a popular non-invasive method to estimate the normal course, location, and extent of white matter tracts, as well as displacement or interruption around a tumor or widening of fiber bundles due to edema or tumor *in vivo* [15]–[18].

DTI relies on diffusion weighted imaging (DWI). The fundamental principle of DWI relies on measuring diffusion properties of water molecules in the human brain. Brownian motion of water molecules is random without any preferential direction of movement, which changes in the presence of structures in the area of interest. The generally disordered diffusion process becomes directional in regions with strongly aligned microstructure, e.g. cell membranes, and the myelin sheath. Thereby, the diffusion process of water molecules is strengthened along the aligned microstructural architecture and hindered in the transverse direction. For each DWI acquisition, a diffusion gradient is applied allowing the measurement of the diffusion process within a specified direction [19].

DTI uses 2^nd^ order tensors to describe the diffusion properties within each voxel. Since the positive and symmetric diffusion tensor requires six coefficients, at least six diffusion weighted images using non-collinear diffusion gradients in addition to one non-diffusion weighted image (b0-image) are indispensable. According to the Stejskal-Tanner equation, the coefficients of the tensors can be determined; the principal eigenvector encodes the dominant diffusion direction, corresponding dominant tissue structure and the mean longitudinal direction of axons in major white matter tracts for each volume element [20], [21].

Until now, several DTI based algorithms have been proposed for reconstructing neuronal pathways in the human brain *in vivo* [22]–[26], generally separated in deterministic and probabilistic methods.

Initial approaches concentrated on deterministic methods [27]–[32]. Based on the assumption that the principle eigenvector, given by the 2^nd^ order tensor, is correlated with the main direction of the underlying fiber structure [33], [34], a path is iteratively calculated, starting at a defined seed point and following the direction parallel to the principle eigenvector at each voxel.

The most commonly implemented method in neuronavigation systems is the so-called tensor deflection algorithm (TEND) [35]. In contrast to traditional streamline tractography based on propagating the streamline in the major eigenvector field, TEND uses the whole diffusion tensor to deflect the incoming tract vector [36] towards the major eigenvector direction with limited deflection curvature, resulting in smoother tracts.

Besides these common techniques, many fiber tractography algorithms have been developed. Merhof et al. presented a connectivity based approach using the A* algorithm for path finding between two selected regions, which was particularly evaluated in case of the language pathways [37]–[39]. Additional global tractography approaches were implemented considering the spatial neighborhood for estimation of fiber orientations [40]–[45].

As opposed to deterministic approaches delivering only one fiber reconstruction per seed point, probabilistic algorithms [44], [46]–[48] consider multiple pathways per seed point and per point along the reconstructed pathways. One probabilistic fiber tracking method has been developed by Kreher et al. [25]. For each voxel ascribed to the fiber bundle, a streamline is propagated through the tensor field. The trajectories are chosen depending on random experiments, in contrast to the trajectory calculation within the deterministic approach. Another probabilistic method is presented by Friman et al. [26] using a Bayesian approach for fiber tracking. On a global level, the probabilities of a connection between two areas in the brain are estimated. On a local level, the probability density functions concerning the fiber orientation are estimated using the Bayes’ theorem.

For clinical purposes, specific white matter tracts have to be selected. After streamline calculation, followed by reducing the resulting fiber sets using include and exclude regions [49], representative 3D objects are calculated as wrapping hulls. The wrapping process is commonly based on a stepwise calculation of bounding curves, such as convex hulls along the set of streamlines [50]. However, the surface is fully dependent on the tracking results and heavily influenced by tracking errors [51]. One example of surface rendering by wrapping is described by Ding et al. [52]. Another approach, directly calculating a 3D volume, has been presented by Merhof et al. [53]. Starting from a predefined seed region, the volume is spread out directionally, taking the shape of the local tensor into account and determining the direction of the growing process.

Alternatively, segmentation algorithms can be used, dividable into different levels. A first class of segmentation algorithms uses scalar anisotropy measures derived from the tensor data and applies routinely used image segmentation methods [54], losing directional information on the underlying structure. More advanced techniques are based on clustering approaches. On a first level, fiber tracts are reconstructed. Subsequent segmentation is performed using pairwise similarity measures, e.g. the Euclidean distance, the ratio of the length for corresponding portions of the fiber to their overall length [55] or by applying normalized cuts after reducing the fibers to a feature vector [56]. Since these depend on previous tractography, a third group of segmentation approaches works directly on the tensor data, without extracting fiber pathways. Using metrics on symmetric positive semi-definite tensor fields, such as the Euclidean metric trace between two tensors [57], [58] or the normalized tensor scalar product [59], traditional segmentation approaches such as spectral clustering [57], [60] or level set methods [58], [59] are applied. Since these methods concentrate more on the segmentation of discrete tensors, rather than on continuous fiber pathways, more sophisticated metrics such as log-Euclidean metric [61], information theoretic metrics [62] or affine-invariant metrics [63]–[65] are used. Locally constrained region based methods [66], [67] use the minimization of an energy function in a probabilistic framework.

Although fiber bundle directions are often well estimated with commonly used fiber tracking techniques, the actual size of the fiber bundles is frequently underestimated, which causes severe problems when fiber bundle reconstruction is integrated into neurosurgical applications [68]. This underestimation of the spatial extent and its tendency to concentrate on the tract center, rather than on the tract borders, can be explained in part with partial volume effects. Partial volume effects cause a decrease in anisotropy due to averaging the diffusion and thereby disturb the main diffusion direction at the white matter tract borders [69].

To visualize the uncertainty of fiber orientation in combination with data of trajectories, boot strapping methods were introduced [70], [71] using multiple repetitions (e.g. nine) of image acquisition, generating a large amount of data sets (e.g. 5000) and finally applying tractography to all of them, providing visitation maps for estimation of confidence and uncertainty. Newer methods, such as wild bootstrapping [72], [73], overcome the prolonged acquisition times for bootstrapping by generating tensor volumes on the basis of tensor fitting and computing the residuals to the fitted model, with similar tractography results like fiber tractography for conventional bootstrapping [74]. Another approach, following a similar idea, has been presented by Hahn et al. [69]. In contrast to repetitions of image acquisition, complex Gaussian noise is added, delivering several data sets with variational noise. Fiber tracking is then performed for all data sets and streamlines are accumulated. Thereby, a widening of aggregation of fibers can be observed with the ability to use the same short image acquisition procedure as for conventional analysis in clinical routine. A new method using a combination of probabilistic fiber tractography and tensor clustering has been suggested by Barbieri et al. [75]. The tractogram provided by probabilistic fiber tractography is used as initial fuzzy segmentation mask, which is iteratively updated according to connectivity information from probabilistic fiber tracking and local tensor clustering. Thereby, the approach incorporates the ability to capture fibers deviating from the main tensor diffusion direction (probabilistic fiber tracking) and the more precise delimitation of bundle borders (tensor clustering).

Due to the still remaining lack of certainty of reliability of the reconstruction methods and the underestimation of fiber bundles using currently approved fiber tractography techniques, in clinical routine the wrapping “hulls” are extended, resulting in so called safety hulls. Nimsky et al. [76] showed that a distance of 5 mm between the boundary of the originally reconstructed object and second surface (hull) is sufficient for the corticospinal tract to avoid neurological deficits. In case of bad data quality or vicinity of tumors and edema, it may be necessary to enlarge this distance.

In this paper, we focus on the reconstruction of large fiber bundles, such as the corticospinal tract. As already shown by Hattingen et al. [77], the deterministic streamline tracking approach significantly depends on the localization of seed regions. Whereas in their study seed region placement in the primary motor areas yields more successful tracking results even with decreased FA values, seed region placement in the cerebral peduncle creates a higher number of fibers tending to be of higher quality. Due to this dependence on seed region placement and to overcome the influence of manual seed region placement, we combine the results of several fiber tracking reconstructions for final reconstruction and visualization. In contrast to the previously described methods based on physically or artificially enlarged data sets, the proposed method deals with a systematic re-seeding for fiber tractography and fiber bundle representation. This approach will be evaluated using software phantoms with modeled anatomical fiber tracts to have ground truth data for comparison.

## Materials and Methods

### Data Acquisition

To evaluate the new approach on seed region independent fiber tract visualization, a software phantom based on the BrainWeb project [78] was used with given ground truth to compare against, modeling part of the left corticospinal tract [79], [80]. To model different qualities of data, the phantom data set was additionally varied with complex Gaussian noise, with reduced signal to noise ratios about 65 (noise 1) and 32 (noise 2), comparable to acquired DTI data on our 3T MRI System (Tim Trio, Siemens, Erlangen, Germany) with signal to noise ratios of around 38.

Additionally, the presented method was also applied to MRI data of two healthy volunteers (27 years old female and 30 years old male), acquired on a 3T MRI System (Tim Trio, Siemens, Erlangen, Germany) at the University of Marburg including a T1-weighted 3D image (3D-Magnetization Prepared Rapid Gradient Echo (MPRAGE): repetition time (TR) 1900 ms, echo time (TE) 2.26 ms, field of view (FoV) 256 mm, matrix 256×256, slice thickness 1 mm, 176 slices, sagittal), a diffusion weighted image data set using single shot echo planar imaging (TR 7800 ms, TE 90 ms, FoV 256 mm, matrix 128×128, slice thickness 2 mm, numbers of excitation 1, b = 1000s/mm^2^, 30 non-collinear diffusion-encoding gradients, voxel size of 2×2×2 mm^3^) and a functional MRI data set using a word generation task (TR 2000 ms, TE 30 ms, FoV 230 mm, matrix 64×64, slice thickness 3.6 mm, voxel size of 3.6×3.6×3.6 mm^3^).

Furthermore, the algorithm was applied to MRI data of a 56 years old male patient with a left precentral anaplastic astrocytoma WHO III and a 65 years old female patient with a left temporo-parietal glioblastoma multiforme WHO IV. The same protocol as for the volunteers was used for data acquisition.

Informed written consent was obtained from both patients before MRI data acquisition, as part of a prospective study on patients with primary brain tumors. Study approval was given by the local ethics committee of the University of Marburg. Informed consent was also obtained from both volunteers, members of our research group, for testing MRI acquisition schemes (in coordination with the local ethics committee).

### Image Analysis

**Figure 1:**
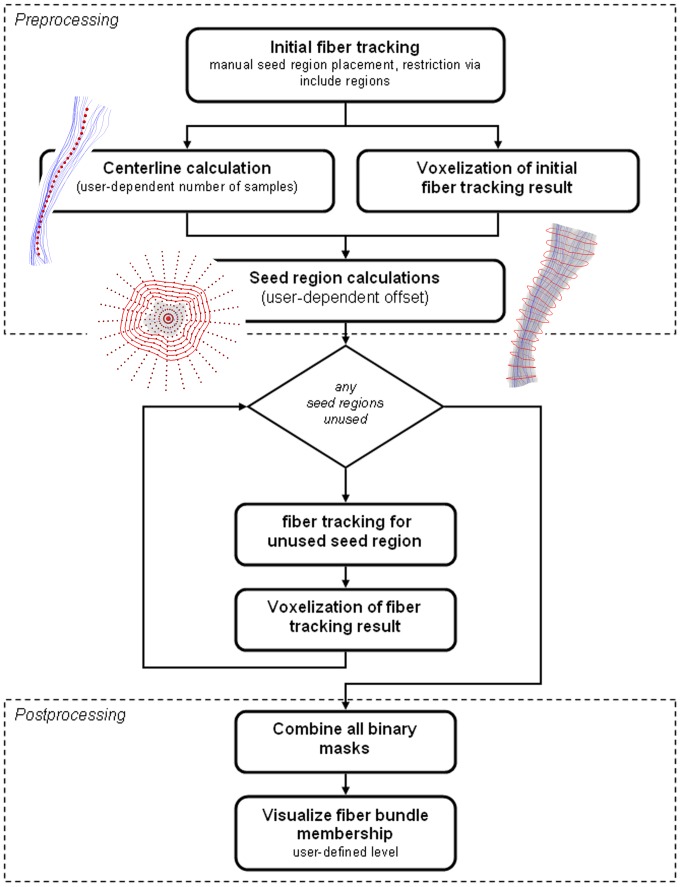
Workflow of the repeated fiber tracking approach.

The procedure is structured into a preprocessing step for seed region calculation, a processing unit for fiber tracking, and a post processing step for object generation. The workflow is summarized and illustrated in Figure 1.

#### Step 1: ROI definition

To set up an initial fiber reconstruction of the desired white matter tract, ROIs are placed manually to define seed and target regions.

In case of the corticospinal tract (and the part of the modeled corticospinal tract in phantom data), the seed region is manually drawn by an experience neurosurgeon outlining the cerebral peduncle in the T1-weighted data set. The second seed region is manually drawn, outlining the precentral gyrus.

As for the arcuate fascicle, activation areas from fMRI data acquisition using a word generation paradigm were used to define Broca’s and Wernicke’s area as seed and target regions. Alternatively, e.g. in case of non-utilizable fMRI results, seed regions were drawn manually outlining the horizontal part of the arcuate fascicle lateral to the corticospinal tract within coronal images (with FA overlay) [81]. The second seed region is drawn manually outlining the descending portion of the arcuate fascicle in the posterior temporal lobe [82].

#### Step 2: Initial fiber tractography and fiber mask generation

Using the defined first ROI as seed region, an initial fiber tract is reconstructed using deterministic fiber tractography within the medical image processing platform NeuroQLab [83] (as well as tensor calculation on a set of DWI data). The initial reconstruction result is then restricted by the application of the second ROI (target) used as include region.

The resulting fiber bundle is mapped to a binary mask where each voxel scores the gray value of 1 if it is crossed or touched by a fiber and of 0 otherwise.

#### Step 3: Centerline calculation

The centerline of the reconstructed fiber bundle is calculated according to Klein et al. [84]. For this purpose, the single streamlines are sampled at *n* points, and each of the *n* centerline points is calculated as the average of the corresponding streamline points.

#### Step 4: Seed region calculation

For each sampled point of the centerline, a plane upright to the centerline’s local direction, given by two consecutive centerline points is calculated. Within each plane, according to the preprocessing step of a previous publication on boundary calculation [85], equally distributed rays are sent out, and the rays are sampled at equally spaced points each. The first point (contour point) of each ray outside of the masked tract volume defines the initial fiber tract outlines. The contour of the resulting generated seed region for the plane is then defined by spline approximation of contour points found for the rays (SCALING 0).

Since the deterministic fiber tractography often underestimates the spatial extent of the tract, seed regions with SCALING 0 are enlarged. Therefore, the contour points are moved outwards along the ray according to the SCALING level, i.e. for SCALING 1 the contour points are moved 1 mm outwards.

#### Step 5: Repeated fiber tractography

The main part relates to the application of the deterministic fiber tracking for all calculated seed regions using the initial seed- and include region as alternative include ROIs. Since the fiber bundle center is well estimated with routinely used algorithms, a combined use of the initial ROIs as include regions would not provide much benefit. The alternative use of the regions allows capturing also areas close to boundaries or branches. After finishing all tracking procedures, each fiber tracking result is assembled into a binary image, as it is also done for the initial tracking result. Finally, all binary masks for the calculated seed regions are combined into one mask including gray values from 0 to *n*, where *n* is the number of seed regions.

#### Step 6: Combination and visualization

For visualization, several objects can be generated by applying a threshold to the final mask image to model the level of fiber bundle membership (FBM) of each voxel. Therefore, different levels are defined. The voxels ascribed to the kernel of the fiber bundle are described by an FBM of 100% to 90% (FBM 90), i.e., 90% or more reconstructions of the fiber bundle include the volume element. In analogy, further objects with lower FBM levels (e.g., FBM 80 = 80% or more reconstructions are included) can be created and displayed as a 3D overlay on the fiber bundle kernel.

For our implementation and evaluation, the medical image prototyping platform MeVisLab (www.mevislab.de) and the neuroimaging prototype NeuroQLab [83] were used. Additional modules and scripting routines were implemented in C++ and Python, respectively. Evaluations were performed on an Intel Core i7-2600K CPU, 3.4 GHz, 16 GB RAM, Windows 7 Professional, SP1.

To evaluate the software phantoms with known courses and extents of the modeled fiber bundles, the Dice Similarity Coefficient (DSC) [86] is used. The DSC is a widely used measure in medical imaging studies to quantify the degree of overlap between two segmented objects A and B, in this case the reference segmentation (ground truth) and the algorithmically computed segmentation result. Since a DSC of 1 describes a perfect match of both segmentations, a DSC of 0 indicates that there is no overlap.

The independent variables “number of seed regions (SEEDS)”, “seed region scaling (SCALING)”, “fiber bundle membership (FBM)” and “image noise (NOISE)” are varied systematically according to Table 1. In total, 1440 different parameterizations were evaluated.

**Table 1:** Parameterization of independent variables.

**Variable**	**Value (Group)**
Image noise level (NOISE)	0(A), 1(B), 2(C)
Number of seed regions (SEEDS)	2(A), 3(B), 5(C), 9(D), 17(E), 33(F), 65(G), 129(H)
Seed region scaling (SCALING) [mm]	0(A), 1(B), 2(C), 3(D), 4(E), 5(F)
Fiber bundle membership (FBM) [%]	10(A),20(B), 30(C), 40(D),50(E),60(F),70(G),80(H),90(I),100(J)

For a comparison with standard procedures, deterministic fiber tractography was performed using the two-ROI-approach, with the first ROI as seed region and the second ROI as target region. In addition, original whole brain tractography, using the whole brain as seed volume, and a variant of whole brain tractography, using the whole set of calculated seed regions as seed volume, were performed. Tractography results were in both cases restricted using the initial seed ROI and include ROI as combined include ROIs (AND).

Statistical analysis was performed in IBM SPSS Statistics 20 for Windows (SPSS Inc., Chicago, Illinois). The level of significance is set to p<0.05. Analysis of variance homogeneities was performed applying the Levene test for each independent variable (NOISE, SEEDS, SCALING, FBM). According to the test results, univariate ANOVA with the post-hoc Tukey-HSD-Test or the Games-Howell-Tests were performed. Comparison of repeated fiber tractography with the deterministic fiber tractography methods was performed using the Wilcoxon-Mann-Whitney-U-test.

Further comparison with probabilistic tractography using the volunteer and patient data was performed within FSL (SMRIB Software Library v. 5.0) [87]–[89] using the Diffusion Toolbox FDT [90], [91] and parameter settings as presented in [91].

## Results

### Results of Standard Deterministic Fiber Tracking

The standard deterministic fiber tracking was applied to three types of phantom data according to the image noise.

The two-ROI-approach took only a few seconds each, as well as the variant of the whole brain tractography and original whole brain tractography.

The two-ROI approach resulted in a DSC of 65.08% ±5.31% (range: 56.99%–73.50%) for noise level 0, for noise level 1 in a DSC of 64.73% ±6.02% (range: 54.85%–74.15%). The tracking procedure applied to the image with noise level 2 resulted in a DSC of 65.91% ±6.42% (range: 57.72%–74.64%).

The adapted version of whole brain tractography also led to an underestimation of the tract volume with a DSC of 67.12% ±0.86% (range: 65.38%–67.59%) for noise level 0, a DSC of 75.10% ±0.28% (range: 74.65%–75.33%) for noise level 1 and a DSC of 72.91% ±0.15% (range: 72.61%–72.99%) for noise level 2. Original whole brain tractography led to an underestimation of the tract volume with a DSC of 67.16% for noise level 0, a DSC of 75.03% for noise level 1 and a DSC of 75.54% for noise level 2.

### Analysis of Repeated Fiber Tracking Results

Univariate ANOVA according to the Levene test was performed for the variables NOISE and SCALING with the post-hoc Tukey-HSD-Test. For SEEDS and FBM, Games-Howell-Tests were applied.

The time of segmentation via repeated tracking procedure differed according to the number of seed regions used for repeated tractography, reaching from a few seconds (less seed regions) to about 3 minutes for the largest number of seed regions.

### Impact of Image Noise (NOISE)

According to the grouping variable NOISE, three subgroups were classified with n = 480 samples for each subgroup.

For group A, repeated fiber tracking delivered DSCs, on the average of 69.27% ±10.56% (range: 41.35%–85.44%), 68.93% ±10.96% (range: 40.50%–85.68%) for group B and 68.82% ±10.75% (range: 41.55%–85.66%) for group C (Table 2). According to univariate ANOVA, the image noise does not significantly affect the quality of fiber bundle segmentation (with the DSC as measure of quality) using the presented method (p = 0.794).

**Table 2:** Dice Similarity Coefficient (DSC) according to group variable NOISE.

**Noise level**	**Mean DSC** **[%]**	**Std. Dev.** **[%]**	**Min. DSC** **[%]**	**Max. DSC** **[%]**
0 (A)	69.27	10.56	41.35	85.44
1 (B)	68.93	10.96	40.50	85.68
2 (C)	68.82	10.75	41.55	85.66

### Impact of Seed Region Scaling (SCALING)

According to the grouping variable SCALING, eight subgroups were classified with n = 185 samples each.

The repeated fiber tracking procedure delivered mean DSCs ranging from 67.41% (group A) to 70.32% (group C), with maximal DSCs of 82.03% to 85.68% (see Table 3).

**Table 3:** Dice Similarity Coefficient (DSC) according to group variable SCALING.

**Value of SCALING**	**Mean DSC [%]**	**Std. Dev. [%]**	**Min. DSC [%]**	**Max. DSC [%]**
0 (A)	67.41	10.57	42.33	82.03
1 (B)	70.13	10.75	44.14	85.00
2 (C)	70.32	10.57	45.19	85.68
3 (D)	69.65	10.47	45.42	85.50
4 (E)	68.76	10.69	45.50	84.76
5 (F)	67.77	11.23	40.50	84.18

Significant differences between subgroups related to SCALING were found using the univariate ANOVA (p = 0.008). Post-hoc analysis with the Tukey-HSD-test showed significant differences between group A and group C (p = 0.035), with significantly improved results for group C. No significant differences were found for all other subgroups. Analysis of homogenous subgroups according to Tukey-HSD delivered two homogeneous subgroups 1 (SCALING 0, 1, 3, 4, 5) and 2 (SCALING 1, 2, 3, 4, 5).

### Impact of Number of Seed Regions (SEEDS)

According to the grouping variable SEEDS, six subgroups were classified each with n = 240 samples each.

The presented procedure resulted in mean DSCs ranging from 65.23% (group A) to 70.41% (group F), with maximal DSCs of 74.64% to 85.68% (see Table 4).

**Table 4:** Dice Similarity Coefficient (DSC) according to group variable SEEDS.

**Value of SEEDS (Group)**	**Mean DSC [%]**	**Std. Dev. [%]**	**Min. DSC [%]**	**Max. DSC [%]**
2 (A)	65.23	5.92	54.85	74.64
3 (B)	67.61	8.21	54.31	79.01
5 (C)	69.45	10.21	48.68	82.99
9 (D)	68.43	11.47	40.50	84.58
17 (E)	70.28	11.47	47.43	85.68
33 (F)	70.41	11.66	44.90	85.24
65 (G)	70.39	12.18	42.87	85.59
129 (H)	70.23	12.33	42.33	85.66

The impact of the independent variable SEEDS was evaluated using the Games-Howell-Test. Highly significant differences (p = 0.000) were found between subgroups A and C, A and E, A and F, A and G, A and H with subgroup A providing significantly worse results. Less significant differences were also found between subgroups A and B (p = 0.038) and subgroups A and D (p = 0.023) also giving significantly worse results for subgroup A in comparison to the other groups. All other pair wise comparisons did not deliver significant differences.

### Impact of Membership Variable (FBM)

According to the grouping variable FBM, ten subgroups were classified with n = 144 samples each.

Mean DSCs ranging from 52.57% to 79.92% were reached with the repeated fiber tracking approach according to FBM classification; with maximal DSCs in the range of 62.32% to 85.68% (see Table 5).

**Table 5:** Dice Similarity Coefficient (DSC) according to group variable FBM.

**Value of FBM (Group)**	**Mean DSC [%]**	**Std. Dev. [%]**	**Min. DSC [%]**	**Max DSC [%]**
10 (A)	65.20	10.50	40.50	82.03
20 (B)	72.89	7.72	48.68	84.27
30 (C)	78.59	6.05	59.87	85.68
40 (D)	79.92	5.20	64.20	85.54
50 (E)	78.01	5.05	64.20	84.01
60 (F)	73.94	6.52	54.85	80.81
70 (G)	67.35	5.38	54.31	75.11
80 (H)	63.86	3.96	54.31	73.08
90 (I)	57.72	2.68	50.38	62.32
100 (J)	52.57	5.73	42.33	62.32

According to Games-Howell-Tests no significant differences were found for subgroups C, D, E (C–D: p = 0.598, D–E: p = 0.054, C–E: p = 0.997), for FBM-subgroups B and F (p = 0.963), subgroups A and G (p = 0.472) and A and H (p = 0.914). All other possible pair wise combinations of FBM-subgroups gave highly significant differences (p = 0.000). Considering Dunnett’s-C-Test, subgroups C, D, E are significantly better (according to DSC) than all other groups; subgroups B and F are superior to subgroups A, G, H, I, J. Subgroup G provided better results than subgroups H, I and J, and subgroups A and H gave better results than groups I and J. Finally, subgroup I provided better results than subgroup J.

**Figure 2:**
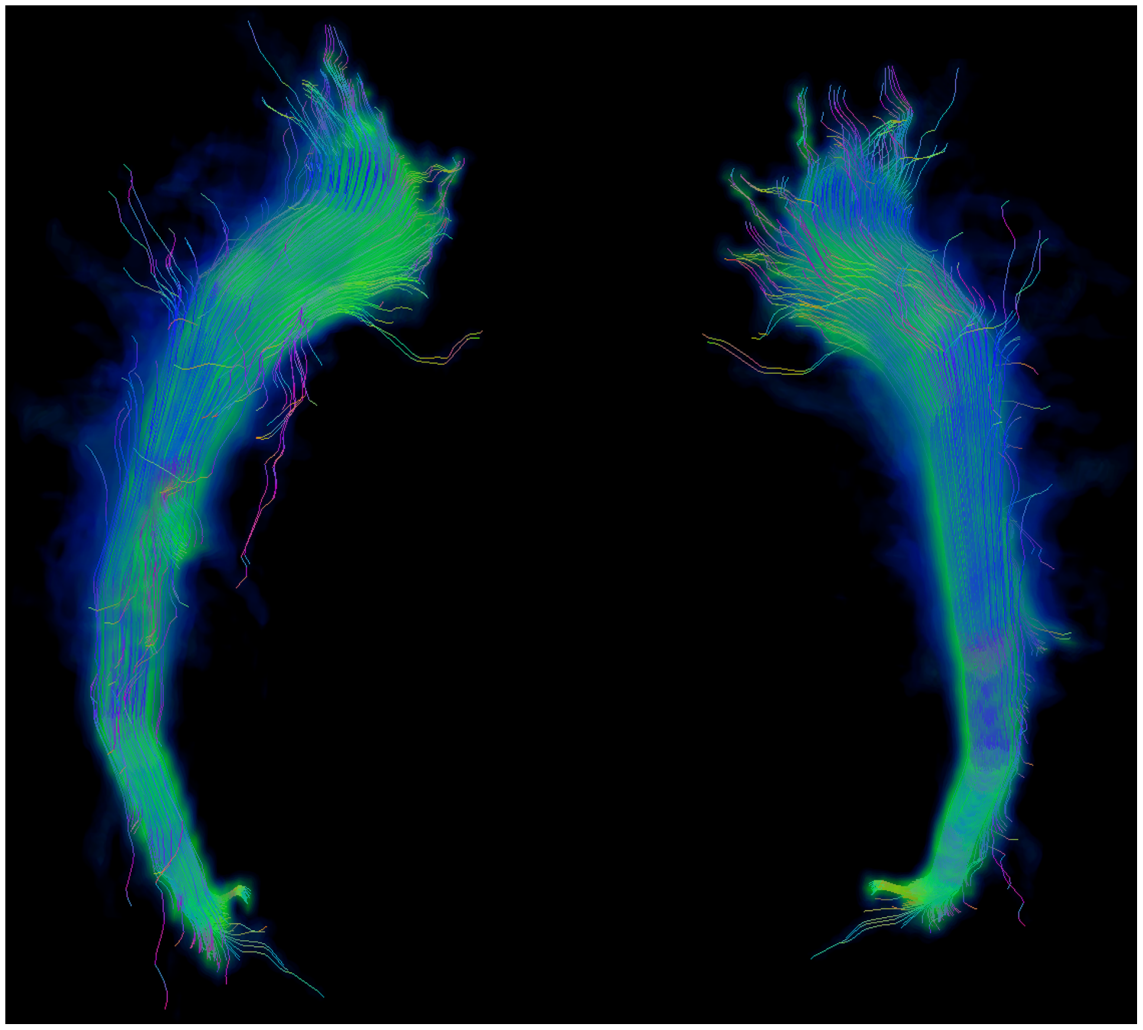
3D view of repeated fiber tracking results. 3D view of results obtained from the repeated tracking approach for an anatomical software phantom with modeled left corticospinal tract, color-coded with green areas indicating regions covered by a large number of fiber tracking applications (high fiber bundle membership) and blue areas indicating regions that are covered by only few fiber tracking results (low fiber bundle membership).

**Figure 3:**
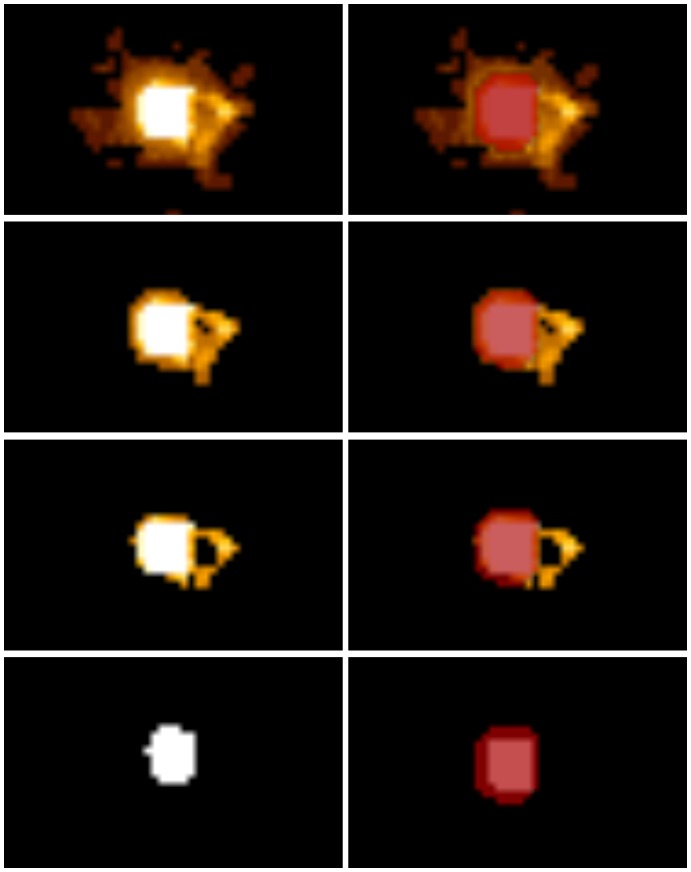
Influence of fiber bundle membership (FBM). Overview of different levels of FBM (left side) with additional overlay of ground truth data in red (right side): FBM 0% (first row), FBM 30% (second row), FBM 50% (third row) and FBM 100% (forth row).

The results of the repeated tracking approach (see Figure 2) do not differ significantly according to the evaluated image noise. The scaling used for seed region generation shows significantly better results for a scaling of 2 mm in contrast to no scaling, providing two homogeneous subgroups with scaling from 1 mm to 5 mm and one subgroup with 0 mm offset and 2 mm to 5 mm offset. Since 2 mm scaling should be preferred over 0 mm scaling, parameterizations of the first subgroup should be considered for good fiber segmentation results. The analysis of the number of seed regions shows significant differences only between the use of 2 seed regions and all other possible evaluated numbers of seed regions; more than 2 seed regions resulted in improved segmentation results. The evaluation of the fiber bundle membership variable delivers significantly different results, best for 30%, 40% and 50% FBM, see Figure 3.

In total, best results were obtained for a scaling of 1 mm to 5 mm, more than 2 seed regions and a fiber bundle membership of 30–50%.

**Figure 4:**
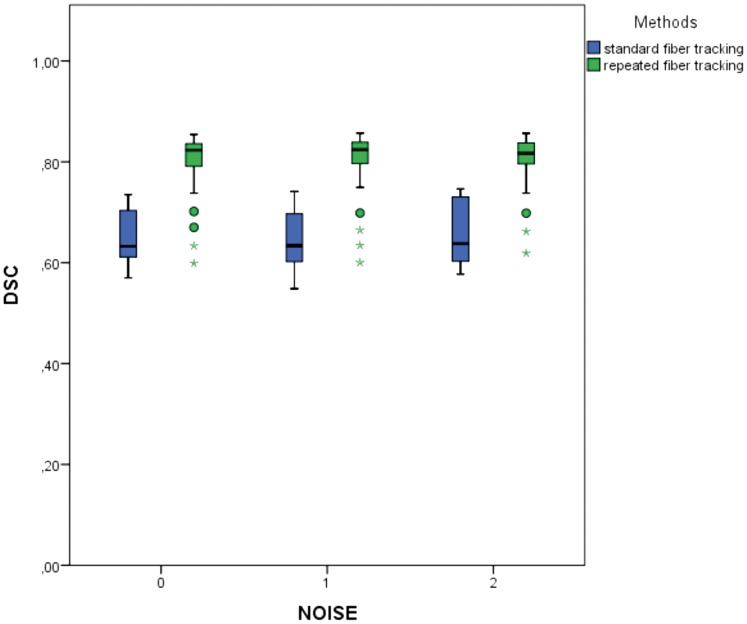
Comparison of standard fiber tracking and repeated fiber tracking. Comparison of results according to the Dice Similarity coefficient (DSC) derived by standard deterministic fiber tracking (in blue) and results of the repeated fiber tracking approach (green) within the group of significantly best parameter settings (n = 315), subdivided by grouping variable NOISE (n = 105).

**Figure 5:**
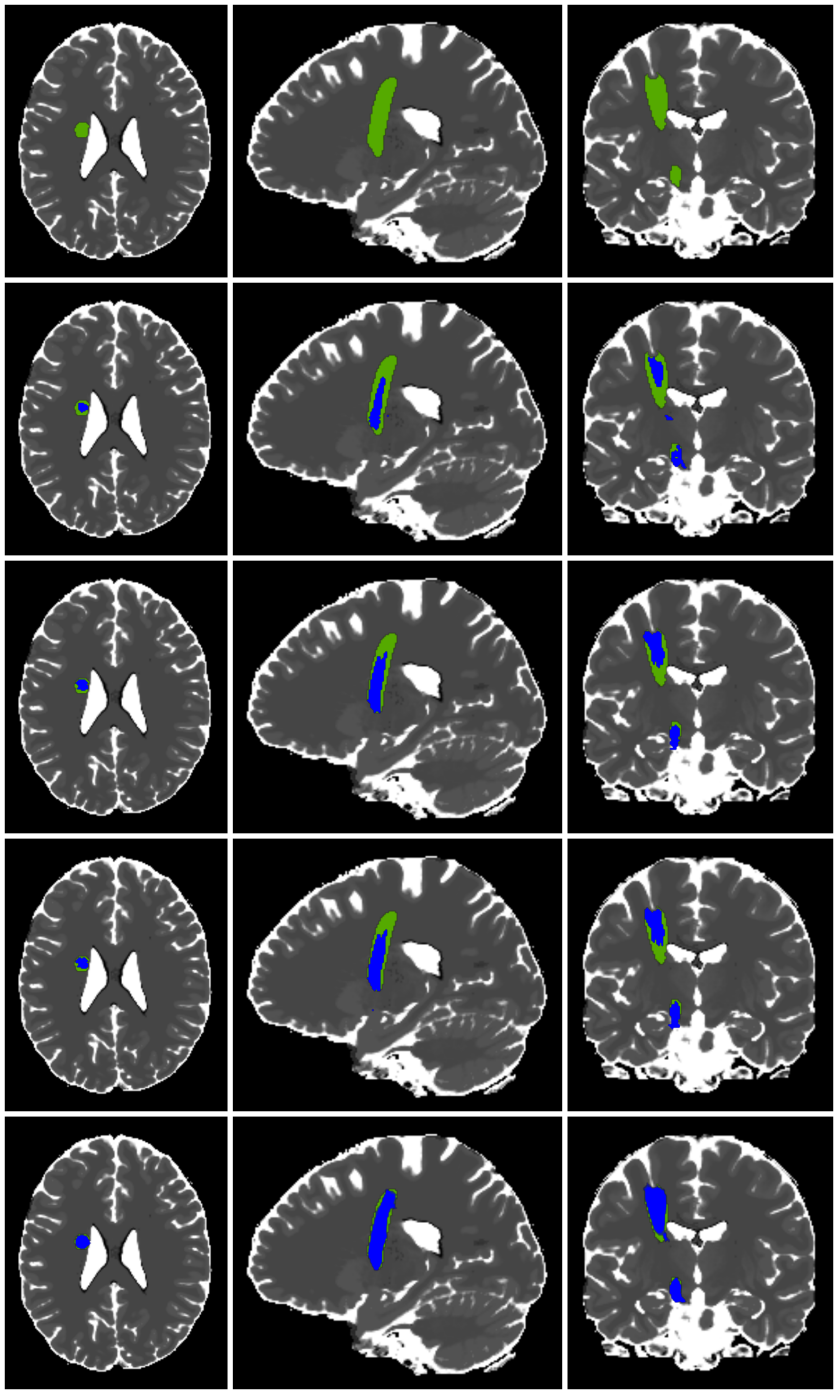
Comparison of two-ROI-approach, whole brain tractography and repeated fiber tracking (example). Comparison of fiber tracking results in blue achieved by the two-ROI-approach (row 2), traditional whole brain tractography (row 3), variant of whole brain tractography (row 4) and the repeated tracking approach (row 5) in axial, sagittal and coronal view within the anatomical phantom data set and underlying modeled ground truth (row 1) in green.

**Figure 6:**
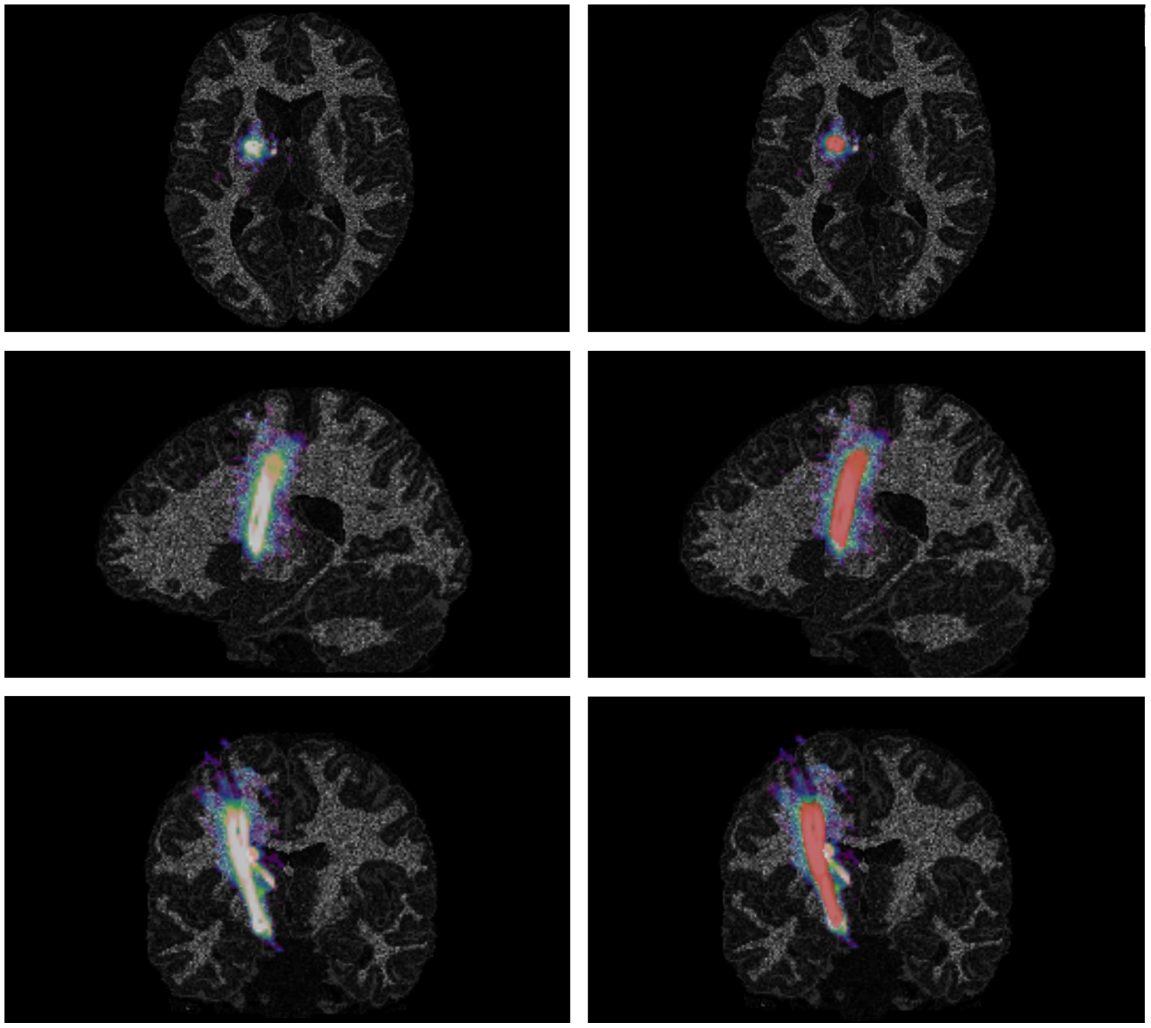
Visualization of results of repeated fiber tracking. Color-coded results of repeated fiber tracking procedure in sagittal, axial and coronal view with summed up fiber tract reconstructions derived from 128 seed region with light areas showing regions of high consensus between the tracking results (classification according to FBM), additional overlay of modeled corticospinal tract as ground truth in red on the right side.

Analysis of this subset of acquired data (n = 105 for each class of NOISE) results in higher values of DSC in correlation to the standard deterministic tracking procedure with only one seed region resulting in DSCs of 65.08% ±5.31% (NOISE 0), 64.73% ±6.02% (NOISE 1) and 65.91% ±6.42% (NOISE 2). The named subset results in mean DSC values of 81.02% ±4.13% (NOSISE 0), 81.32% ±4.22% (NOISE 1) and 80.99% ±3.81% (NOISE 2), see Table 6 and Figure 4. Comparing the subgroups of repeated fiber tracking with deterministic fiber tracking procedures used by default, the standard procedures differed significantly for all noise levels (p = 0.000) according to Wilcoxon-Mann-Whitney-U-test from the achieved results of the presented method. Therefore, the presented approach can be considered as a promising technique to improve the quality of fiber bundle segmentation significantly, see Figures 5 and 6.

**Table 6:** Dice Similarity Coefficients (DSC) for best parameterizations of the repeated fiber tracking approach.

	NOISE 0			
	Repeated FT	two-ROI-FT	Whole brain FT	Whole brain FT
			(variant)	(original)
Mean DSC [%]	81.02	65.08	67.12	67.16
Std. Dev. [%]	4.13	5.31	0.86	
Min. DSC [%]	59.87	56.99	65.38	
Max. DSC [%]	85.44	73.50	67.46	
	**NOISE 1**			
	**Repeated FT**	**two-ROI-FT**	**Whole brain FT**	**Whole brain FT**
			**(variant)**	**(original)**
Mean DSC [%]	81.32	64.73	75.10	75.03
Std. Dev. [%]	4.22	6.02	0.28	
Min. DSC [%]	60.03	54.85	74,65	
Max. DSC [%]	85.68	74.15	75,33	
	**NOISE 2**			
	**Repeated FT**	**two-ROI-FT**	**Whole brain FT**	**Whole brain FT**
			**(variant)**	**(original)**
Mean DSC [%]	80.99	65.91	72.91	75.54
Std. Dev. [%]	3.81	6.42	0.15	
Min. DSC [%]	61.88	57.72	72,61	
Max. DSC [%]	85.66	74.64	72,99	

1 FT: fiber tractography.

With the resulting information about suitable parameterization to improve fiber tractography results and the noise independent behavior, the presented method was applied to healthy volunteer's data and to two patient data sets with intracerebral gliomas. The corticospinal tract was reconstructed for the leg area on the left and right side in case of volunteer data and on the left side in case of the patient data set including the left precentral glioma. Besides, the arcuate fascicle was reconstructed in case of both volunteer data sets and in case of the patient data set including the left temporo-parietal glioma. For repeated fiber tractography, a seed region scaling of 2 mm, 128 generated seed regions and a fiber bundle membership of 30% to 50% was used.

**Figure 7:**
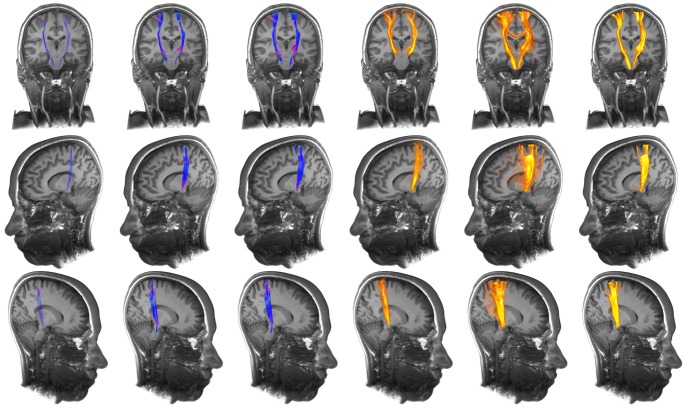
Application of repeated fiber tracking on healthy volunteer data: corticospinal tract. Comparison (from left to right) of the results from standard deterministic fiber tracking, variant of whole brain fiber tracking, whole brain fiber tracking, probabilistic fiber tractography, unfiltered results of the repeated fiber tracking method and filtered results of the repeated fiber tracking method with a fiber bundle membership of 50% using a seed region scaling of 2 mm and 128 generated seed regions.

**Figure 8:**
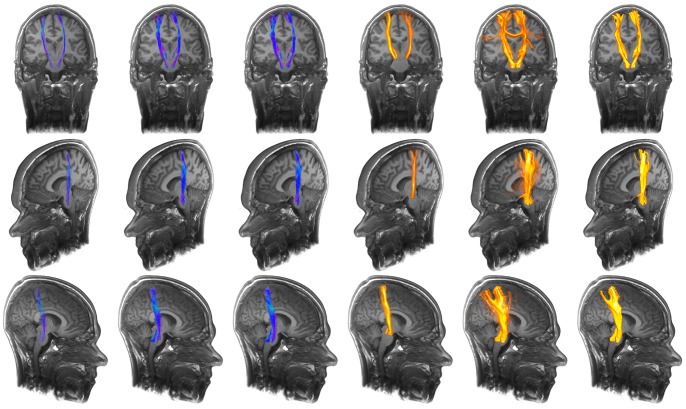
Application of repeated fiber tracking on healthy volunteer data: corticospinal tract. Comparison (from left to right) of the results from standard deterministic fiber tracking, variant of whole brain fiber tracking, whole brain fiber tracking, probabilistic fiber tractography, unfiltered results of the repeated fiber tracking method and filtered results of the repeated fiber tracking method with a fiber bundle membership of 50% using a seed region scaling of 2 mm and 128 generated seed regions.

**Figure 9:**
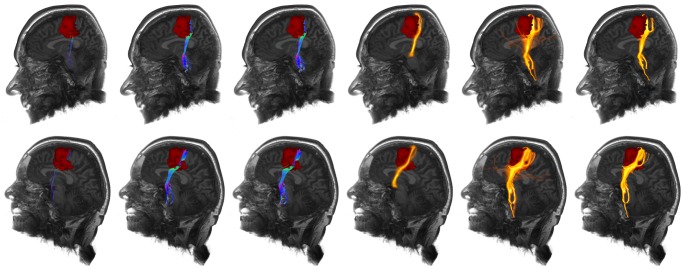
Application of repeated fiber tracking on patient data with a left precentral glioma: corticospinal tract. Comparison (from left to right) of the results from standard deterministic fiber tracking, variant of whole brain fiber tracking, whole brain fiber tracking, probabilistic fiber tractography, unfiltered results of the repeated fiber tracking method and filtered results of the repeated fiber tracking method with a fiber bundle membership of 50% using a seed region scaling of 2 mm and 128 generated seed regions, tumor segmented manually in red.

**Figure 10:**
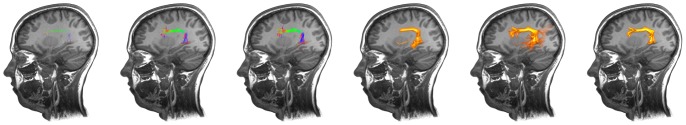
Application of repeated fiber tracking on healthy volunteer data: arcuate fascicle. Comparison (from left to right) of the results from standard deterministic fiber tracking, variant of whole brain fiber tracking, whole brain fiber tracking, probabilistic fiber tractography, unfiltered results of the repeated fiber tracking method and filtered results of the repeated fiber tracking method with a fiber bundle membership of 50% using a seed region scaling of 2 mm and 128 generated seed regions.

**Figure 11:**
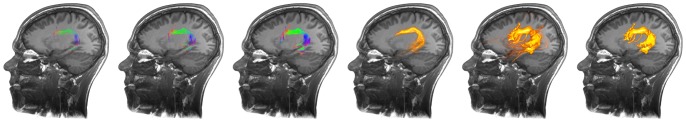
Application of repeated fiber tracking on healthy volunteer data: arcuate fascicle. Comparison (from left to right) of the results from standard deterministic fiber tracking, variant of whole brain fiber tracking, whole brain fiber tracking, probabilistic fiber tractography, unfiltered results of the repeated fiber tracking method and filtered results of the repeated fiber tracking method with a fiber bundle membership of 50% using a seed region scaling of 2 mm and 128 generated seed regions.

**Figure 12:**
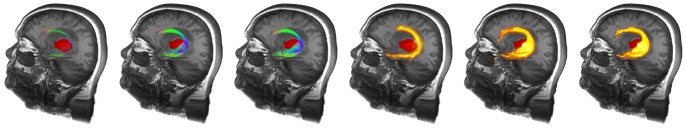
Application of repeated fiber tracking on patient data with a left temporo-parietal glioma: arcuate fascicle. Comparison (from left to right) of the results from standard deterministic fiber tracking, variant of whole brain fiber tracking, whole brain fiber tracking, probabilistic fiber tractography, unfiltered results of the repeated fiber tracking method and filtered results of the repeated fiber tracking method with a fiber bundle membership of 50% using a seed region scaling of 2 mm and 128 generated seed regions, tumor segmented manually in red.

A comparison of the repeated fiber tractography approach (without and with application of FBM), the two-ROI-approach, the whole brain tractography approach, its variant and additionally probabilistic fiber tractography (connectivity analysis) is presented in Figure 7 and 8 for part of the corticospinal tract encoding motor function for the lower extremities, in two healthy volunteers. Furthermore, tractography was applied to a patient data set, with a precentral anaplastic astrocytoma WHO III. Results are shown in Figure 9. Further comparison is presented for the arcuate fascicle in Figures 10 and 11 in case of healthy volunteers and in case of a patient data set with a temporo-parietal glioblastoma multiforme WHO IV. Results are shown in Figure 12.

## Discussion

In all cases, application of the repeated fiber tractography approach led to widened fiber bundle segmentation in comparison to results of the two-ROI-approach and improved results of the whole brain fiber tracking approach in concordance with previous tests on the software phantom data. Probabilistic fiber tractography (within FSL) reconstructs only a subset of the corticospinal tract similar to the other methods. Probabilistic fiber tractography thereby took approximately 6 hours (mainly for preprocessing with BEDPOSTX resulting in a distribution of diffusion parameters for each voxel). Two-ROI-approach and whole brain fiber tractography took only a few seconds, the repeated fiber tracking approximately 3 minutes.

For application in the clinical routine, the use of fiber tractography requires short data acquisition times due to patient compliance, workflow efficiency, and also fast reconstruction techniques for segmentation and especially intraoperative update of functional information. Since DTI data acquisition is possible with short acquisition times in the range of 4–5 minutes on a 3T MRI system and approximately 8 minutes on a 1.5T MRI system (with the previous described setting), the fiber tractography algorithms based on DTI data sets have been tested extensively and deterministic streamline fiber tractography is available in most surgical planning stations. Intensive clinical evaluation has been performed for the corticospinal tract to estimate accuracy of fiber tracking, still longing for more accuracy, and thereby for increased tumor volume reduction [68], [69], [76].

The presented method initially uses the clinically widespread used tensor deflection algorithm based on DTI, combining the result of different automatically initiated applications, similar to previously published methods like the application of bootstrapping [70] or wild bootstrapping [73] or the application of variational noise for uncertainty estimation [69]. In contrast to the bootstrapping technique, the method uses only one acquired data set and thereby keeps the time requirements of clinical usage. In contrast to wild bootstrapping with a need for a model to fit to and calculate residuals, repeated fiber tracking can easily be adapted to other models and model-free approaches for fiber reconstruction [74]. Additionally, no changes of image quality have to be performed for repeated application of fiber tractography. All approaches mentioned above concentrate on the fiber boundary, with repeated measurement using different data sets, noise varied measurements, or re-seeding using the basic data set.

In contrast to probabilistic fiber tractography methods, that deliver a connectivity map, direct thresholding can be applied for fiber bundle segmentation based on the accumulated fiber tracking results. In case of probabilistic tractography, the connectivity score is distance dependent, requiring an additional re-seeding for final fiber bundle segmentation and application in neurosurgical practice, increasing the processing time.

Up to now, as DTI is still the routinely applied model, fiber tractography algorithms based on the 2^nd^ order tensor model are used in clinical practice. However, there are many challenging topics arising due to the use of DTI. With its restricted 2^nd^ order tensor model, assuming Gaussian distributed diffusion, voxels with multi-fiber populations cannot be represented correctly, resulting in an inability to resolve crossing or kissing fibers. Even fanning fiber tracts are hard to resolve with this assumption [92], for example making it difficult to reconstruct the whole corticospinal tract on the basis of DTI and streamline tractography. Since approximately one third of voxels in the brain contain more than one fiber population [91], different approaches for description of diffusion functions (e.g., different model, model-free approaches) have to be found. Advanced techniques like high angular resolution diffusion imaging (HARDI) [93] or Q-Ball imaging (QBI) [94] offer opportunities for the application of advanced diffusion functions to overcome the drawbacks of the restricted tensor model provided by DTI. Up to now, these techniques are not widely used in clinical applications due to their long acquisition times, high hardware-performance requirements and processing times. Acquisition times for scanning protocols using 128 diffusion encoding gradients are up to 20 minutes on a 3T MRI system, and nearly 40 minutes on a 1.5T MRI system, which are most commonly available. Thus, until now these techniques are of lower feasibility in clinical practice. Nevertheless, they will be increasingly suitable for clinical evaluation and clinical use.

Due to the modular structure of the repeated fiber tracking framework, the used diffusion model “diffusion tensor” as well as the basic deterministic streamline tractography algorithm can easily be replaced by other techniques resulting in a streamline representation. Approaches resulting in a 3D volume representation of the fiber bundle, like the volume growing approach [53], need an alternative preprocessing step for centerline calculation, skeletonizing the volume to its centerline.

In the future, alternative approaches will be included for repeated fiber tracking, also based on advanced imaging techniques, if image acquisition and processing could be performed within adequate times (e.g. HARDI with compressed sensing techniques) for example resolving the fanning of the corticospinal tract, or white matter tracts that can hardly be visualized in clinical routine.

For theoretical purposes, time consuming HARDI techniques and advanced fiber tractography algorithms are suitable, however not efficiently applicable in clinical routine.

Our study group currently works on tractography on the basis of currently published theoretical approaches using compressed sensing techniques for reconstruction of HARDI signals using sparse data [95], [96] in comparison to traditional HARDI acquisition. Initial experience on the reconstruction of the language pathways using HARDI signals with compressed sensing techniques in comparison to DTI related reconstruction based on the same DTI data sets (30 gradient diffusion encoding directions), delivered promising results [38]. Until now, HARDI using compressed sensing is not implemented on a commercially available software platform. Thus, integration of these data into neuronavigation systems remains a future perspective. Finally, we aim at the integration in the repeated fiber tractography framework.

The presented approach concentrates on the commonly reconstructed part of the corticospinal tract, as large white matter tract in the human brain, which has been extensively examined and evaluated for clinical purposes. Since the repeated fiber tracking approach delivers promising results for better estimation of the spatial extent of the tract, also in the vicinity of intraaxial tumors (Figure 8), where anatomy is distorted and diffusion patterns might be changed, it will also be adapted to other relevant white matter tracts. A first impression is given by the reconstruction of the arcuate fascicle (Figure 9) in consistence with results obtained from the corticospinal tract. Furthermore, this approach will be tested for other large white matter tracts such as the optic radiation on basis of the developing advanced methods and evaluated clinically.

### Conclusions

We presented a new fast approach for the reconstruction of fiber bundles in diffusion weighted imaging data using automatically calculated reconstruction along initial tracking results for the desired white matter tract. Evaluation on an anatomical software phantom shows that the presented method is able to improve the segmentation quality of white matter tracts with adequate processing times significantly in contrast to the standard tensor deflection (two-ROI-approach and whole brain tractography approaches), which is routinely used in most clinical settings.
